# Numerical exploration of forced convection hydromagnetic hyperbolic tangent nanofluid flow over a permeable wedge with melting heat transfer

**DOI:** 10.1038/s41598-023-30656-2

**Published:** 2023-03-02

**Authors:** Mehari Fentahun Endalew, Subharthi Sarkar

**Affiliations:** 1 Department of Mathematics, Debre Tabor University, Debre Tabor, Ethiopia; 2 Department of Mathematics, Banwarilal Bhalotia College, Asansol, India

**Keywords:** Applied mathematics, Computational science

## Abstract

In this communication, the joint impacts of the process of melting as well as wedge angle entity on hydromagnetic hyperbolic tangent nanofluid flow owing to permeable wedge-shaped surface in the incidence of suspended nanoparticles along with radiation, Soret and Dufour numbers are scrutinized. The mathematical model which represents the system consists of a system of highly non-linear coupled partial differential equations. These equations are solved using a finite-difference-based MATLAB solver which implements the Lobatto IIIa collocation formula and is fourth-order accurate. Further, the comparison of computed results is carried out with the previously reported articles and outstanding conformity is recorded. Emerged physical entities affecting the bearings of tangent hyperbolic MHD nanofluid velocity, distribution of temperature, and concentration of nanoparticles are visualized in graphs. In another line, shearing stress, the surface gradient of heat transfer, and volumetric rate of concentration are recorded in tabular form. Most interestingly, momentum boundary layer thickness and thicknesses of thermal as well as solutal boundary layers enhance with an increment of Weissenberg number. Moreover, an increment on tangent hyperbolic nanofluid velocity and decrement on the thickness of momentum boundary layer is visualized for the increment of numerical values of power-law index entity, which can determine the behavior of shear-thinning fluids.This study has applications for coating materials used in chemical engineering, such as strong paints, aerosol manufacturing, and thermal treatment of water-soluble solutions.

## Introduction

Hyperbolic tangent fluid is a class of non-Newtonian fluids demonstrating shear thinning behavior (see Ref.^1,2^). Mathematical model of this fluid has distinct advantage compared with other classes of non-Newtonian fluids that includes computational effortlessness, easiness of understanding and the strength of physical interpretations. Fluids such as ketchup, lava, whipped cream, paints and blood can be considered as tangent hyperbolic fluids. Nowadays, extensive research works on tangent hyperbolic fluids have been explored by many researchers across the world under the considerations of various circumstances. Among those few are: Ibrahim^3^, Patil et al.^4^, Atif et al.^5^ and Ibrahim and Tezera^6^.

The non-Newtonian Magnetohydrodynamics (MHD) is a class of fluid dynamics which takes part in a notable role in industrial as well as engineering applications including power production, thermal protection, pumps and the like. In common sense, it devotes with the mutual interaction of electrically conducting (but non-magnetic) fluids under the influence of applied magnetic field. Recently, many researches on hydromagnetic flows in different geometrical configuration are conducted. For example, a closed form solution on time dependent MHD flow due to oscillating plate embedded by homogeneous permeable medium has discussed by Endalew et al.^7^. Hydromagnetic rotating Maxwell fluid flow due to unidirectional stretching surface is reported by Ramaiah et al.^8^. Further, Muhammmad et al.^9^ have performed hydromagnetic viscous fluid due to a curve-shaped surface.

Nanofluids are fluids with suspending nanoparticles. The concept of this innovative class of fluid was proposed by Choi and Estman^10^. They tried to fill the primary gaps of low thermally conductive fluids in the improvement of energy-efficient heat transfer fluids which have significant applications in several industrial and engineering fields. It is well noted that nanofluids are important to enhance heat transfer coefficient compared with that of its base fluids. They have significant numerous applications involving heat transfer as well as industrial processes, transportation, nuclear reactors, electronics, food, biomedicine, and detergents. Nanofluids have notable applications in solar thermal engineering systems as discussed by Mahian et al.^11^. Review of experimental results, based on theory, about heat transfer abilities of nanofluids was presented by Lomascolo et al.^12^.

The influences of heat-mass transports in joint form through a porous medium have a notable great applications in modern industrial and technological aspects across the world. Mustafa et al.^13^ preformed the joint impacts of mass-heat transfer in the time-dependent squeezing flow between parallel plates. In addition, a phenomena which yields the change of phase of substances from solid state to liquid state by means of heat transfer is commonly known as melting process. There are so many practical interests of melting heat transfer in every day activities. Among those few are; storing thermal energy, heating and cooling process, unfreezing of grounds and so on. Very recently, the investigation of joint impacts of mass and heat transfer on hydromagnetic nanofluid flowing towards non linear stretching Riga plate was performed by Vaidya et al.^14^. Analysis of heat transfer in cylindrical polar system with magnetic field is investigated by Jalili et al.^15^. Moreover, Sarkar and Endalew^16^ have investigated influences of melting on hydromagnetic Casson nanofluid flow due to a wedge implanted through permeable medium containing variable permeability. Impact of melting process on micropolar fluid flow due to a stagnation point is studied by Adegbie et al.^17^. Moreover, important investigations regarding heat and mass transfer with combination of nanofluids are found in Refs.^18–23^.

Thermal radiation is detected as heat or light, and it occupies an intermediate wavelengths. That is the radiant emission depends on temperature means it is function of temperature by its nature. It is very important in current technological and industrial aspects. Utilization of sun’s radiation as an energy source on earth is one of the most important practical applications of thermal radiation in real life. Nowadays, thermal radiation influences in various fluids with the imposition of different conditions is studied by different researchers such as Amjad et al.^24^, Endalew and Nayak^25^, Endalew et al.^7^ and Pattnaik et al.^26^. In general, thermal analysis of different fluids with respect to various geometrical configurations has beeen investigated by Jalili et al.^27–29^.

The investigation of flows past wedge-shaped bodies have received rapt attention of researchers all over the world owing to its applications in different areas including engineering, science, technology, etc. Physically, wedge problem is a problem dealing with flows in which non-parallel to the plates. Formerly, this kind of problem was described by Falkner- Skan equation which describes external flow of laminar boundary layer forms. Mass and heat transports on fluid flow over a wedge gets numerous uses in insulation and thermal engineering, agriculture, polymer industry, solar power absorbs, aerospace engineering, crude oil extraction etc. Hassan et al.^30^ reported the flow problem of boundary layer for hybrid non-Newtonian nanofluid due to a wedge in motion. Moreover, several researchers studied flows over a wedge-shaped bodies with mixed or Robin type boundary conditions such as Newtonian heating or melting heat transfer. Among those researchers few are: Ahmad et al.^31^, Ishak et al.^32^, Hossain et al.^33^ and Sarkar and Endalew^16^. Khan et al.^34^ investigated impacts of magnetized radiative flow of sutterby nanofluid subjected to convectively heated wedge.

A material which contains a solid matrix with an interconnected pores or voids is commonly termed as a porous medium (see Ref.^35^. Some common natural and human-made examples of porous medium are sponges, cement, soil, biological tissues, bones and so on. The vital role of these interconnected voids or pores is to permit flow of fluid through the material. Its characteristics differ relying on the arrangement, size, pores or voids shape, porosity and compositions of material itself. Thin film flow through porous medium is investigated by Endalew and Sarakar^36^. Investigation of flow of second grade fluid through microchannel containing porous material inside it under the influences of dual-phase-lag (DPL) heat-mass transfer is performed by Sarkar et al.^37^. Moreover, researches such as Endalew et al.^38^, Chinedu et al.^39^ and Endalew and Sarkar^40^ described the fluid behavior in porous materials.

However, the investigation of magnetohydrodynamic flow of a hyperbolic tangent nanofluid past a permeable wedge with melting heat transfer is yet to be carried out even though its wide industrial applications is well understood from the above discussion. Therefore, the major target of the current research work is to explore the various physical entities influencing time-independent two-dimensional forced convective hyperbolic tangent hydromagnetic nanoparticle suspending fluid flow due to a permeable wedge along with melting heat transfer. In addition, we also consider the effects of diffusion-thermo as well as thermo-diffusion in order to gain a wider perspective for this kind of flow problem. The fundamental equations describing the tangent hyperbolic fluid flow problem are changed into solvable ordinary differential equations by introducing the concept of similarity transformation. Similar solutions for the transformed ordinary differential equations are obtained using the bvp4c subroutine of MATLAB. The behavior of pertinent physical entities affecting hyperbolic tangent nanofluid velocity, temperature distribution and volumetric concentration is explored in graphical illustrations and briefly explained. Moreover, commonly known but the most important coefficients such as shearing stress, rate of heat transfer as well as rate of mass transfer are documented in table and discussed in detail in terms of physical rationale.

**Figure 1:**
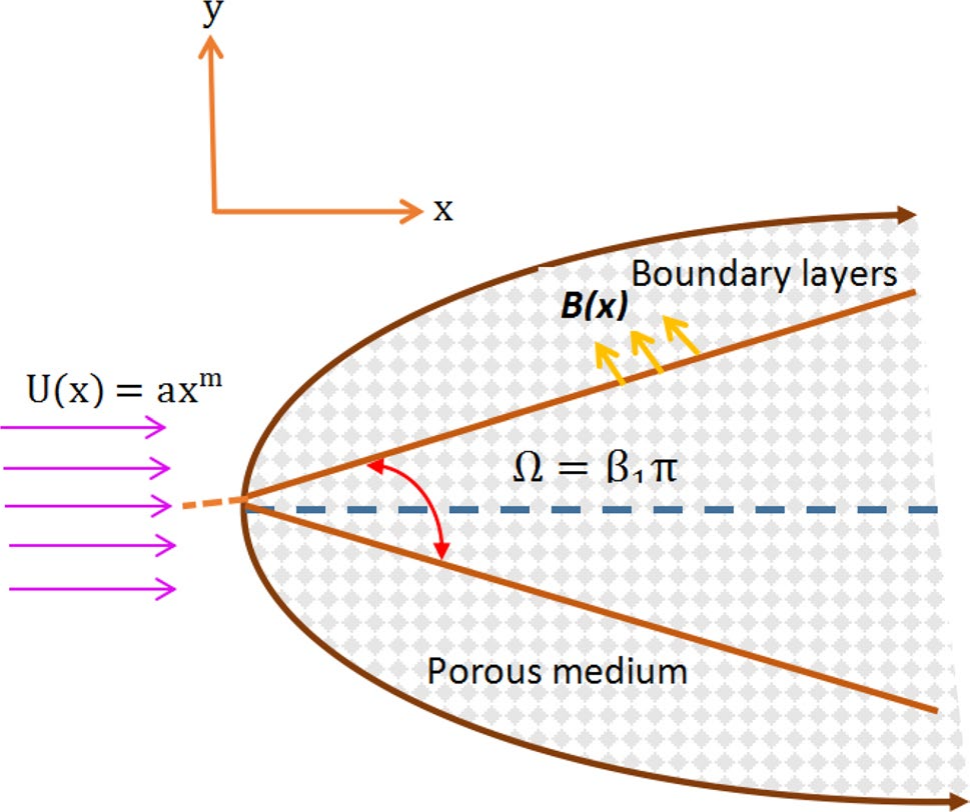
Geometrical illustration of the model.

## Mathematical foundation of the problem

In this article, *2D* time-independent forced convection incompressible, radiating, electrically conducting tangent hyperbolic nanoparticle suspending fluid flow over a permeable wedge with inclusion of melting heat transfer is scrutinized. Here, incidences of diffusion-thermo as well as thermo-diffusion are incorporated too. $C_w$ denotes volumetric fraction of concentration and $T_m$ stands for temperature of surface caused by melting process. The nanofluid velocity which is outside of boundary layer is assumed to be $U(x)=ax^m$, here *a* is positive real constant (see Refs.^16,32^). Further, the variable magnetic field can be expressed as $B(x)=B_ox^{\left( \frac{m-1}{2}\right) }$, here, the constant magnetic $B_o$ is applied perpendicular to the wedge walls (see^16^). Moreover, the permeability is supposed to be variable and expressed in the form of $K(x)= K'x^{-(m-1)}$, here, $K'$ symbolizes the constant permeability of the medium. The wedge angle parameter can be expressed as $m=\frac{\beta _1}{2-\beta _1}$ such that $\beta _1=\frac{\Omega }{\pi }$ (^16^). Here, $\Omega$ symbolizes the wedge angle and $\beta _1$ represents the Hartree pressure gradient. The value of *m* is assumed that $0\le m\le 1$. However, for the horizontal plate its value becomes $m=0$ and for the vertical plate it is specified as $m=1$. $T_{\infty }$ symbolizes the nanofluid temperature away from the surface. $C_{\infty }$ describes the volumetric fraction of nanoparticle far from the surface. Here, $T_m>T_{\infty }$ as well as $T_m>T_o$.

The constitutive representation of hyperbolic tangent fluid equation can be expressed in the form:

$$\begin{aligned} \varvec{\tau }=\left[ \left( \mu _\infty +\mu _0\right) tanh(\Gamma {\dot{\Omega }})^n+\mu _\infty \right] {\dot{\Omega }} \end{aligned}$$

Here, $\varvec{\tau },\Gamma$ and *n* symbolize the extra stress tensor, material constant of time as well as power law index, respectively. $\mu _0$ represents the null shear rate viscosity, $\mu _\infty$ stands for the infinite shear rate viscosity and ${\dot{\Omega }}$ can be expressed as:

$$\begin{aligned} {\dot{\Omega }}=\sqrt{\frac{1}{2}\sum _{m}^{}\sum _{k} {\dot{\Omega }}_{mk}{\dot{\Omega }}_{km}}= \sqrt{\frac{1}{2}{\textbf{A}}}. \end{aligned}$$

Note that as power law index equal to one ,i.e $n=1$, the considered fluid is immediately reduced to Newtonian fluid. From the above expression, ${\textbf{A}}$ symbolizes the second order strain rate tensor invariant. It can be defined as:

$$\begin{aligned} {\textbf{A}}=\frac{1}{2}tr\left[ \left( grad {\varvec{V}}\right) ^{{\varvec{T}}}+\left( grad {\varvec{V}}\right) \right] ^2. \end{aligned}$$

In the above expression ${\varvec{T}}$ stands for the Cauchy stress tensor and **V** represents the fluid velocity. Assume that $\mu _\infty =0$, and we are focusing on the hyperbolic tangent fluid which represents shear thinning behavior when $\Gamma {\dot{\Omega }}<1$ and an expression for $\varvec{\tau }$ reduces to,

$$\begin{aligned} \varvec{\tau }=\mu _0{\dot{\Omega }} \left[ (\Gamma {\dot{\Omega }})^n\right] = \mu _0{\dot{\Omega }}\left[ (\Gamma {\dot{\Omega }} -1+1)^n\right] =\mu _0{\dot{\Omega }} \left[ -1+n(\Gamma {\dot{\Omega }}+1)\right] . \end{aligned}$$

Having aforementioned assumptions, the fundamental equations governing the hyperbolic tangent hydromagnetic nanofluid flow problem can be given as^1,3,5^:

### Equation of continuity

1 $$\begin{aligned} \frac{\partial v}{\partial y}+\frac{\partial u}{\partial x} =0, \end{aligned}$$

### Equation of momentum

2 $$\begin{aligned} v\frac{\partial u}{\partial y}+u\frac{\partial u}{\partial x}=U(x)\frac{\partial U(x)}{\partial x}+\nu \left[ (1-n)+\sqrt{2}n \Gamma \left( \frac{\partial u}{\partial y}\right) \right] \frac{\partial ^2 u}{\partial y^2} +\left( \frac{\sigma B^2(x)}{\rho _f} +\frac{\nu }{K(x)}\right) \left( U(x)-u\right) , \end{aligned}$$

### Equation of energy

3 $$\begin{aligned} v\frac{\partial T}{\partial y}+u\frac{\partial T}{\partial x}= \alpha \frac{\partial ^2 T}{\partial y^2} +\frac{\kappa _TD_B}{C_sC_p}\frac{\partial ^2 C}{\partial y^2} -\frac{1}{\rho C_p}\frac{\partial q_r}{\partial y} +\Lambda \left\{ D_B\frac{\partial C}{\partial y} \frac{\partial T}{\partial y}+ \frac{D_T}{T_\infty }\left( \frac{\partial T}{\partial y} \right) ^2\right\} , \end{aligned}$$

### Equation of concentration

4 $$\begin{aligned} v\frac{\partial C}{\partial y}+u\frac{\partial C}{\partial x}=\frac{\kappa _T D_B}{T_m}\frac{\partial ^2 T}{\partial y^2}+ \frac{D_T}{T_\infty }\frac{\partial ^2 T}{\partial y^2}+D_B\frac{\partial ^2 C}{\partial y^2}, \end{aligned}$$

From the aforementioned equations, (*u*, *v*) symbolize the components of velocity, *C* symbolizes the volumetric fraction of concentration in dimensional form, *T* stands for the temperature in dimensional form, $D_T$ is the coefficient of thermophoresis diffusion, $D_B$ stands for the Brownian diffusion, $\Lambda =\frac{(\rho c)_p}{(\rho c)_f}$ defines the quotient of heat capacity of nanofluid to the common fluid, $\nu$ symbolizes the viscosity of nanofluid, $\alpha =\frac{k}{(\rho c)_f}$ symbolizes the thermal diffusivity of nanofluid, *n* specifies the power law index of the fluid, $\sigma$ stands for the electrical conductivity, and $\kappa _T$ represents the ratio of thermal diffusion.

### Boundary conditions

The appropriate boundary conditions to evaluate the fundamental equations governing tangent hyperbolic nanoparticle suspending fluid flow is expressed as (^16,41^):

5a $$\begin{aligned}{} & {} u=0,T=T_m,v=0,k\frac{\partial T}{\partial y}=\rho v(x,0)\left[ C_s(T_m-T_o)+\lambda \right] , C=C_w \text{ at } y=0, \end{aligned}$$

5b $$\begin{aligned}{} & {} v=0, u\rightarrow U(x),C \rightarrow C_{\infty },T\rightarrow T_\infty \text{ as } y \rightarrow \infty . \end{aligned}$$

In the above expressions, $\lambda , k,T_o,C_s$ and $\rho$ symbolize the latent heat of the nanofluid, thermal conductivity of the nanofluid, temperature of solid surface, solid surface heat capacity and density, respectively. The main focus of boundary conditions in this research is dealing with the concept of melting process (see 5a). Physically speaking, melting point is the point which materials converts from the solid state to the liquid state. This process has multiple physical and industrial applications as mentioned in the introduction section of this research work.

Following Ref.^42^, an optically thick fluid is considered here, the radiation heat flux can be expressed using Rosseland approximation and written as:

6 $$\begin{aligned} q_r=-\frac{4\sigma ^*}{\kappa ^*}\frac{ \partial T^4}{\partial y}, \end{aligned}$$

where, $\kappa ^*$ stands for the absorption constant and $\sigma ^*$ specifies the Stefan-Boltzmann constant. By applying Taylor series expansion in $T^4$ about $T_\infty$ with exclusion of higher order terms of this series, we are left with the following equation:

7 $$\begin{aligned} T^4\cong -3T^4_\infty +4T^3_\infty T. \end{aligned}$$

Substitution Eqs. (6) and (7), in (3) yields,

8 $$\begin{aligned} v\frac{\partial T}{\partial y}+u\frac{\partial T}{\partial x}= \left( \alpha +\frac{16\sigma ^*T^2_\infty }{3\rho C_p\kappa ^*}\right) \frac{\partial ^2 T}{\partial y^2}+\frac{\kappa _TD_B}{C_sC_p}\frac{\partial ^2 C}{\partial y^2} +\Lambda \left\{ D_B\frac{\partial C}{\partial y}\frac{\partial T}{\partial y}+ \frac{D_T}{T_\infty }\left( \frac{\partial T}{\partial y} \right) ^2\right\} . \end{aligned}$$

The stream function denoted by $\psi (x,y)$ is given in the form of:

9 $$\begin{aligned} v=-\frac{\partial \psi }{\partial x},u=\frac{\partial \psi }{\partial y}. \end{aligned}$$

In order to make the dimensionless fundamental equations governing the flow problem, the following dimensionless and similarity variables are introduced:

10 $$\begin{aligned} \psi =f(\eta )\sqrt{\frac{2x\nu U(x)}{m+1}}, \theta (\eta )=\frac{T-T_\infty }{T_m-T_\infty }, \eta =\sqrt{\frac{(m+1)U(x)}{2x\nu }}y, \phi (\eta )=\frac{C-C_\infty }{C_w-C_\infty }. \end{aligned}$$

The continuity Eq. (1) is satisfied for the above defined stream function. Equations (2–4) are transformed into the following coupled highly non-linear ordinary differential equations by implementing similarity transformation:

11 $$\begin{aligned}{} & {} \left( 1-n+nWif''\right) f'''+ff''+\left( \frac{1}{K_p}+ M\right) (1-f')+\frac{2m}{1+m}\left( 1-f'^2\right) =0, \end{aligned}$$

12 $$\begin{aligned}{} & {} \left( 1+\frac{4Rd}{3}\right) \theta ''+\left\{ D_4\phi ''+Nb\phi '\theta '+Nt\theta '^2+f\theta '\right\} Pr=0, \end{aligned}$$

13 $$\begin{aligned}{} & {} \phi ''+PrLe\phi 'f+\theta ''\left( PrLeSr+\frac{Nt}{Nb}\right) =0. \end{aligned}$$

The subsequent transformed boundary conditions are givens as follows:

14a $$\begin{aligned}{} & {} f'(\eta )=0, \theta (\eta )=1,B\theta '(\eta ) +P_rf(\eta )=0,\phi (\eta )=1 \text{ at } \eta =0,\end{aligned}$$

14b $$\begin{aligned}{} & {} f'(\eta )\rightarrow 1,\theta (\eta )\rightarrow 0,\phi (\eta )\rightarrow 0 \text{ as } \eta \rightarrow \infty , \end{aligned}$$

The flow parameters are defined as:

15 $$\begin{aligned} M&=\frac{2\sigma B^2_o}{\rho _f a(m+1)},K_p=\frac{aK'(m+1)}{2\nu },Pr=\frac{\nu }{\alpha },B= \frac{C_f(T_m-T_o)}{\lambda +C_s(T_m-T_o)},\nonumber \\ Nt&=\frac{D_T(T_m-T_\infty )\Lambda }{\nu T_\infty },Nb=\frac{D_B(C_w-C_\infty ) \Lambda }{\nu },Le=\frac{\alpha }{D_B}, Rd=\frac{4\sigma ^*T^3_\infty }{\kappa ^*\kappa }\\ Wi&=\sqrt{\frac{\Gamma ^2(m+1)U^3(x)}{\nu x}},D_4=\frac{\kappa _TD_B(C_w-C_\infty )}{C_sC_p(T_m-T_\infty )},Sr=\frac{\kappa _TD_B (T_m-T_\infty )}{T_m\nu (C_w-C_\infty )},\nonumber \end{aligned}$$

where, $\theta , f', \phi ,Le,Pr,Nb, Wi,Nt,D_4,Sr,M,Rd$ and $K_p$ are the temperature in dimensionless form, velocity in non-dimensional form, volumetric fraction of concentration in dimensionless form, Lewis and Prandtl numbers, correspondingly, Brownian diffusion, Weissenberg number, parameter of thermophoresis, Dufour nimber, Soret number, magnetic field, thermal radiation and permeability of the medium, respectively.

Mathematically melting can be expressed as $B= \frac{C_pf(T_m-T_o)}{\lambda +C_s (T_m-T_o)}$. Here, Stefan numbers assigned for liquid and solid states and can be defined as $\frac{C_pf(T_m-T_o)}{\lambda }$ and $\frac{C_s(T_m-T_o)}{\lambda }$, respectively.

Moreover, the dimensionless form of the surface shearing stress, heat transfer rate as well as volumetric fractional rate of nanoparticles can be expressed as follows:

16 $$\begin{aligned} \sqrt{Re_x}C_f=\left( 1-n\right) f''(0) +\frac{n}{2}Wi\left( f''(0)\right) ^2, \frac{Nu}{\sqrt{Re_x}}=-\left( 1+Rd\frac{4}{3}\right) \theta '(0), \frac{Sh}{\sqrt{Re_x}}=-\phi '(0), \end{aligned}$$

where $Re_x$ specifies the local Reynolds number, $C_f$ denotes the shearing stress, *Nu* is surface gradient of heat transfer and *Sh* surface gradient of volumetric concentration.

## Implementation of numerical technique

The numerical solutions for fundamental equations governing the flow problem (11–13) subjected to their corresponding boundary conditions (14a–14b) are obtained using MATLAB. In essence, we have used the bvp4c solver which uses the method of finite difference. This is an implementation of three-stage Lobatto IIIa formula (see Refs.^3,6,43^). This formula is a collocation type and its polynomial affords a $C^1$-continuous fourth-order accurate solution which is uniform in the given interval. Control of error and selection of mesh depend on the residual of the continuous solution. Here, the solution starts with an initial guess provided at an initial mesh points and changes step-size in order to find the specified accuracy. This method is a convenient and easy to use and capable of solving fairly sophisticated problems. The algorithm relies on an iteration structure for solving nonlinear systems of equations. The solver determines a numerical solution by solving a global system of algebraic equations resulting from the boundary conditions, and the collocation conditions imposed on all the subintervals. The solver then estimates the error of the numerical solution on each subinterval. If the solution does not satisfy the tolerance criteria, the solver adapts the mesh and repeats the process. We must provide the points of the initial mesh, as well as an initial approximation of the solution at the mesh points.

In order to solve transformed ordinary differential Eqs. 11–13 subjected with transformed boundary condition 14a–14b numerically, bvp4c routine of MATLAB is used. To reduce these equations into first order ordinary differential equations, one can set in the form:

17 $$\begin{aligned} \left. \begin{aligned} f=f_1,\\ f'=f_2,\\ f''=f_3,\\ \theta =f_4,\\ \theta ^{\prime }=f_5,\\ \phi =f_6,\\ \phi '=f_7,\\ f'_3=\frac{-1}{1-n+nWif_3}\left( \frac{2m}{m+1}(1-f^2_2)+f_1f_3+(M+K^{-1}_P)(1-f_2)\right) ,\\ f'_5=\frac{-Pr}{1+\frac{4}{3}Rd} \left( D_4f'_7+Nbf_5f_7+Ntf^2_5+f_1f_5\right) ,\\ f'_7=-\left( \frac{Nt}{Nb}+PrLeSr\right) f'_5-PrLef_1f_7, \end{aligned}\right\} \end{aligned}$$

with boundary conditions:

18 $$\begin{aligned} \left. \begin{aligned} f_2(\eta )=0,f_4(\eta )=1, Bf_5(\eta )+P_rf_1(\eta )=0,f_6(\eta )=1 \text{ at } \eta =0,\\ f_2(\eta )\rightarrow 1,f_4(\eta )\rightarrow 0,f_6(\eta )\rightarrow 0 \text{ as } \eta \rightarrow \infty . \end{aligned}\right\} \end{aligned}$$

### Validity of numerical scheme

**Figure 2:**
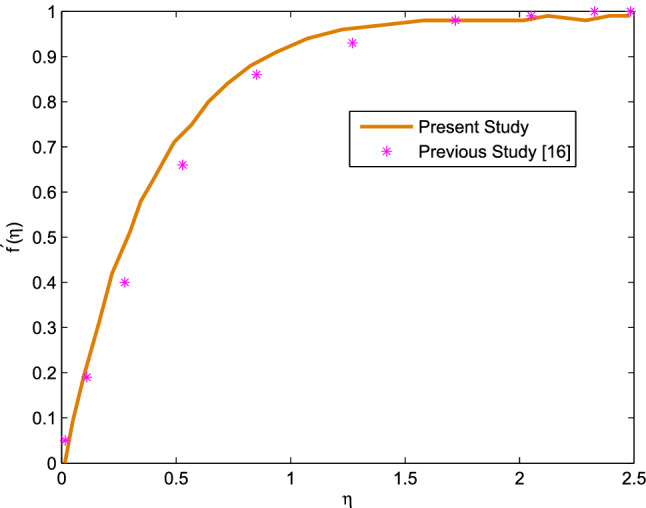
Validity of the results.

In order to make sure the accuracy of the present MATLAB code, the values of shearing stress are replicated from the existing article reported by Sarkar and Endalew^16^. In this line, we set the values of governing parameters as $n=-0.1, Wi=0$ to reduce the current skin friction Eq. (16) in to the existing skin friction equation existed by Sarkar and Endalew^16^ in equation (13) with $\beta =10$. In addition to this Dufour number, Soret number and thermal radiation are omitted. Then we have implemented the same code and we have found results in an excellent agreement as it can be shown in table 1. In addition, by making $n=Wi=D4=Sr=Rd=0$, and extracting some points from Fig. 2 of Sarkar and Endalew^16^, we have plotted the graph with the same code and it is found in an excellent agreement as shown in Fig. 2.

Similarly, by omitting parameters *n*, *Wi*, *m*, *Le*, *Sr*, *D*4, *M*, *Kp*, *Rd*, *Nb* and *Nt*, the error analysis of shearing stress with existing exact solution of Ishak et al. (Fig. 1 in Ref.^41^) corresponding to $\epsilon =0$ is made and the result found in an excellent agreement as recorded in Table 2.

**Table 1:** Comparison of shearing stress ($f''(0)$) with existing results.

*m*	*Le*	*B*	Sarkar and Endalew^16^	Present work
0.5	1	1	1.74873	1.74874
0.6	1	1	1.77412	1.77413
0.5	2	1	1.75051	1.75051
0.5	3	1	1.75133	1.75133
0.5	1	2	1.66148	1.66148
0.5	1	3	1.60535	1.60535

**Table 2:** Error analysis of shearing stress $f''(0)$ (for $Pr=1$) against Ishak et al.^41^ (Fig. 1) corresponding with $\epsilon =0$.

*B*	Present results	Ishak et al.^41^	Absolute error $(x_i)$	Mean Error $({\overline{x}})$	Standard Deviation $(S_x)$
0 (Blasius)	0.47115	0.4696	0.00155	${\overline{x}}=\frac{1}{n}\sum \limits _{i=1}^{n=4}x_i =0.00454$	$S_x=\sqrt{\frac{1}{n-1}\sum \limits _{i=1}^{n=4}\left( x_i-{\overline{x}}\right) ^2}=0.0017263$
1	0.28256	0.2774	0.00516		
2	0.20718	0.20323	0.00395		
3	0.1656	0.1581	0.0075		

## Results and discussion

In this article, various emerging pertinent physical entities affecting tangent nanofluid velocity, temperature distribution and volumetric concentration are carefully analyzed and disclosed in Figs. 3, 4, 5, 6, 7, 8, 9, 10, 11, 12, 13, 14, 15, 16, 17, 18, 19, 20, 21, 22, 23, 24, 25, 26, 27 and 28. In addition, the notable coefficients such as shearing stress, the rate of surface heat transfer and volumetric rate of mass transfer are portrayed in Table 3. Similar numerical solutions are obtained by implementing BVP4C subroutine of MATLAB. Also, results are authenticated based on the existing publications reported by Sarkar and Endalew^16^ and Ishak et al.^41^ as explored in Tables 1 and 2. In this research work, the default values for the emerging physical parameters are fixed as: $n=Wi=m=Le=0.5,Sr=D4=M=Kp=0.1,Rd=B=1,Nb=Nt=0.2, Pr=3$, unless otherwise mentioned.

**Figure 3:**
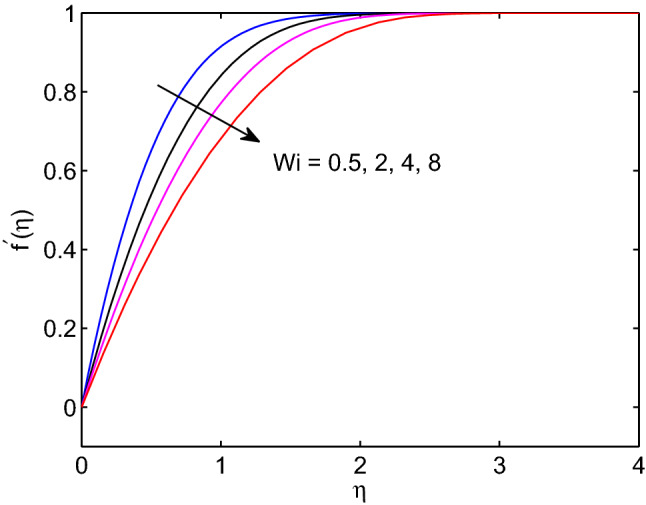
Weissenberg number effects on velocity profiles.

**Figure 4:**
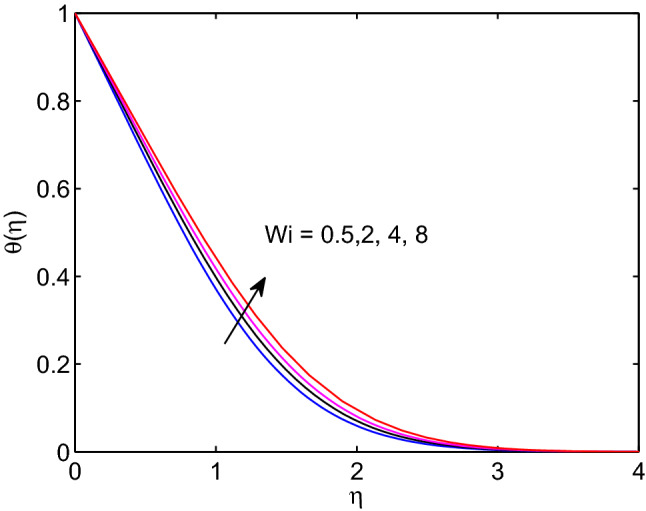
Weissenberg number effects on temperature profiles.

**Figure 5:**
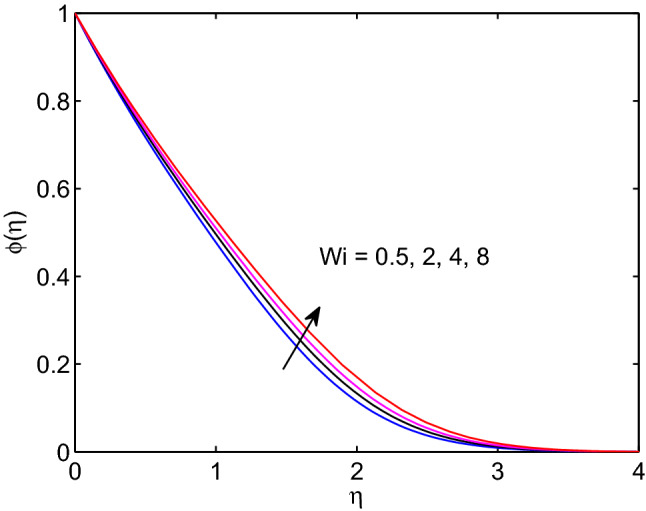
Weissenberg number effects on volumetric concentration profiles.

Figures 3, 4, 5 show effects of Weissenberg number on tangent hyperbolic nanofluid velocity, temperature distribution and volumetric concentration along with their corresponding boundary layers. As it can be seen form the given figures, hyperbolic tangent nanofluid velocity reduces with the increase of Weissenberg number whereas both tangent hyperbolic nanofluid temperature and volumetric concentration increase with increase of this number. From the physical viewpoint, Weissenberg number can be expressed as the ratio of elasticity to the fluid viscosity. Owing to this physical fact, velocity boundary layer thickness, solutal and thermal boundary layer thicknesses expand with the rise of Weissenberg number as because of high Weissenberg number fluids provide low resistivity thus taking a longer time to meet free stream velocity, temperature and volumetric concentration.

The impact of power law index *n* on hyperbolic tangent nanofluid flow is revealed in Fig. 9. Mainly, the power law index *n* describes the shear thinning fluids by its nature. When we increase numerical values of *n*, hyperbolic tangent fluid velocity can accelerate easily. Because of this the tangent hyperbolic nanoparticle suspending fluid flow elevates with the increment of the power law index entity.

**Figure 6:**
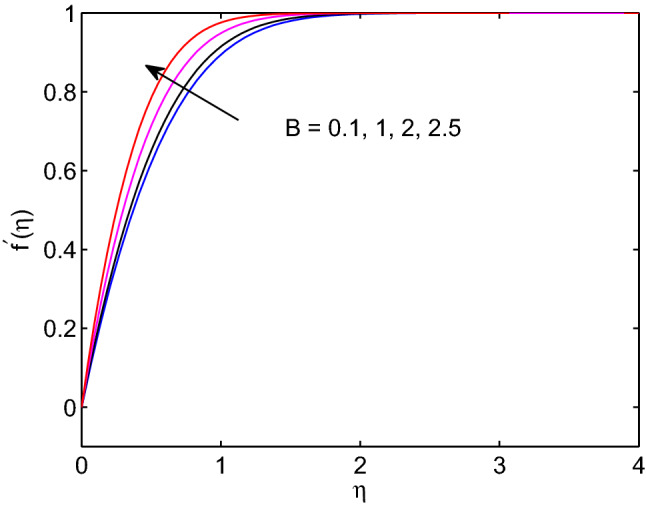
Melting heat transfer effects on velocity profiles.

**Figure 7:**
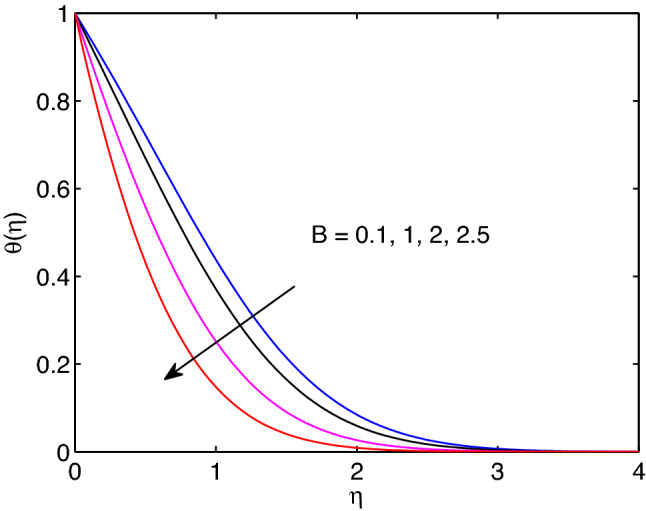
Melting heat transfer effects on temperature profiles.

**Figure 8:**
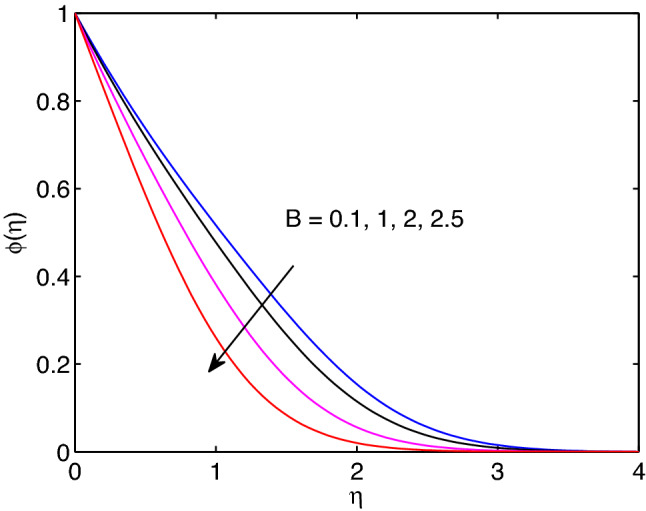
Melting heat transfer effects on volumetric concentration profiles.

Melting is a process of changing from solid state to liquid state by means of heating process. During this process, energy draws from the layers of fluid close to the surface through the boundary layer. Hence, imposing melting at boundaries has a liability to diminish gradients of both temperature and volumetric concentration as shown in Figs. 7 and 8. However, it has a tendency to enhance the fluid velocity as it can be seen in Fig. 6.

**Figure 9:**
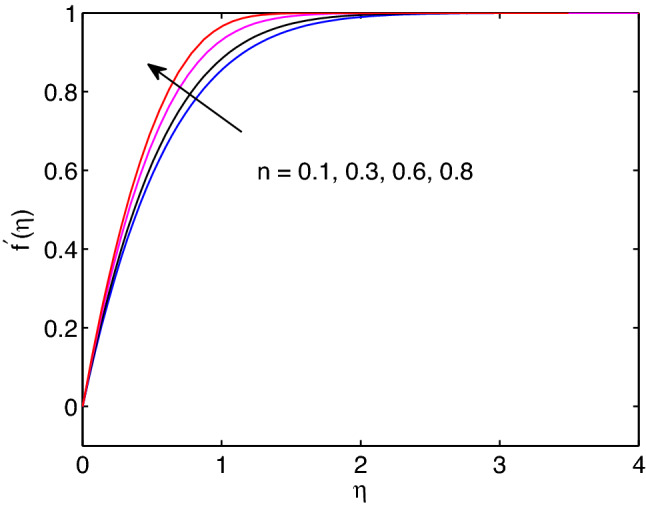
Effects of power law index on velocity profiles.

**Figure 10:**
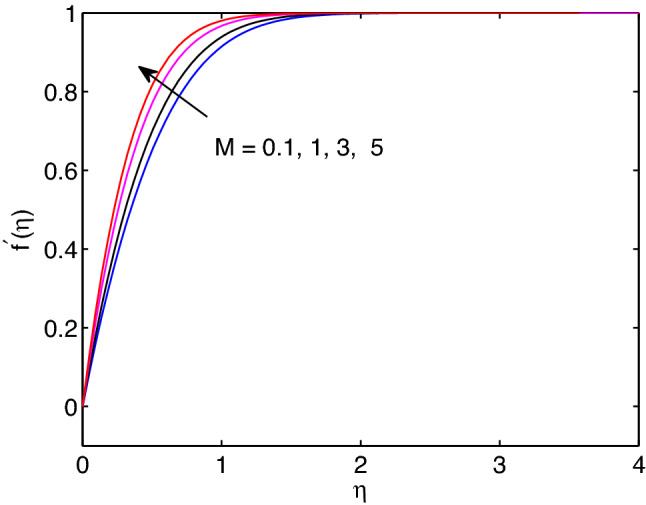
Magnetic field effects on velocity profiles.

**Figure 11:**
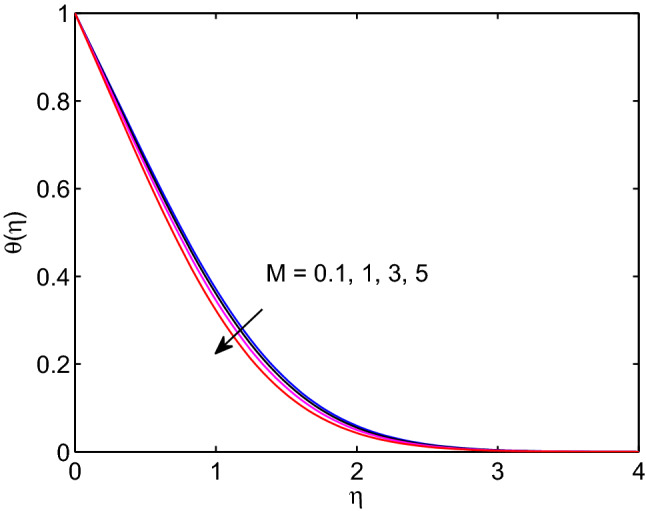
Magnetic field effects on temperature profile.

**Figure 12:**
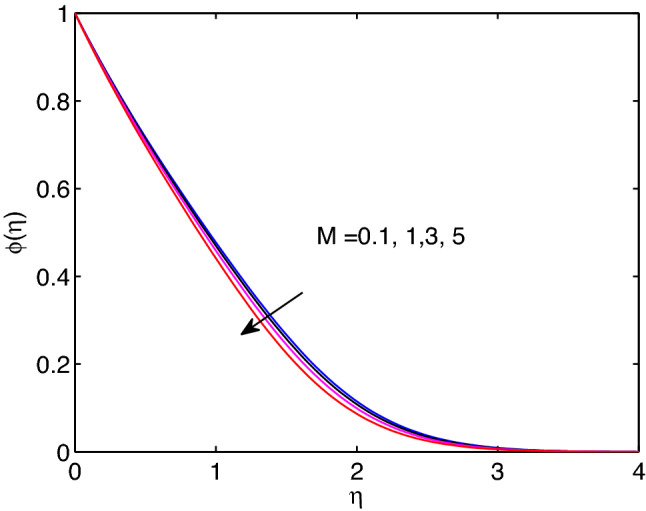
Magnetic field effects on volumetric concentration profiles.

The entity of magnetic field effects on hyperbolic tangent nanofuid velocity, the distribution of temperature and volumetric fraction fluid concentration are captured in Figs. 10, 11 and 12. Usual features of magnetic field is highly influencing the fluid flow due to the direct relationship with Lorentz force. However, the contrary result to this feature of magnetic field is observed in our investigation as it can be shown in Fig. 10. When we look at the right side of Eq. (2), which holds magnetic field, a body force occurs with positive sign. Here, this body force generated from the fact of physics i.e., $u<U(x)$. That is the external or free stream velocity dominates the flow system through the boundary layer. In fact, the hyperbolic tangent nanoparticle suspending fluid velocity approaches to external (free stream) velocity in advance as the strength of magnetic field parameter enhances. This means the magnitude of tangent hyperbolic nanofluid velocity expands with the rise of magnetic field entity. One can see from the Figs. 11 and 12, both temperature distribution and volumetric concentration diminish with the boost of magnetic field parameter. Thicknesses of momentum, thermal and volumetric concentration boundary layers diminish with the increment of magnetic field entity as well.

**Figure 13:**
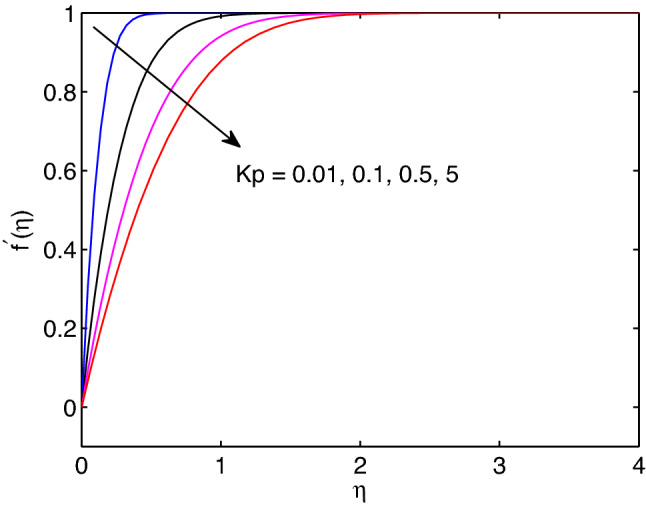
Permeability effects on velocity profiles.

**Figure 14:**
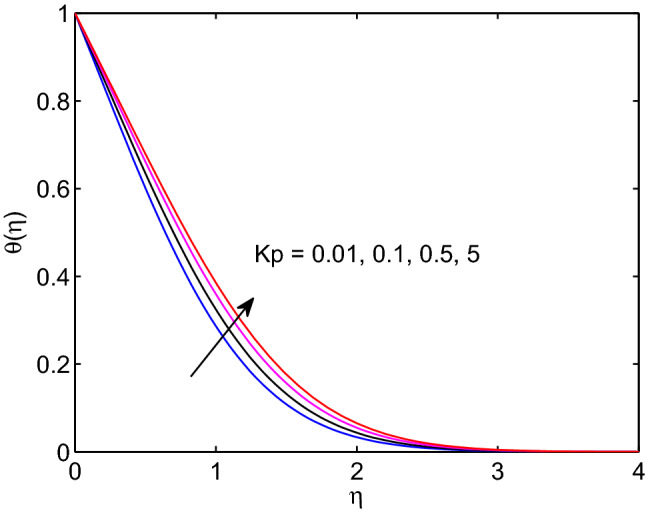
Permeability effects on temperature profiles.

**Figure 15:**
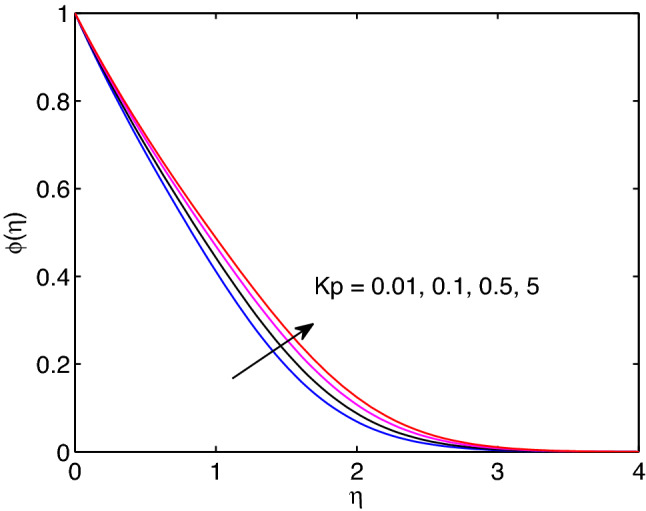
Permeability effects on volumetric concentration profiles.

The effects of permeability of medium on velocity of hyperbolic tangent fluid, nanofluid temperature and nanoparticle concentration are revealed in Figs. 13, 14 and 15. Inhere, one can see that momentum, thermal and solutal boundary layers thicknesses expand with the expansion of permeability of the medium. However, the hyperbolic tangent fluid velocity diminishes with the enlargement of permeability of medium as shown in Fig. 13. This is due to hyperbolic tangent fluid velocity meets external velocity in a while for the larger values of permeability. The opposite approach of permeability effect on hyperbolic tangent nanofluid flow is analogous to the earlier impacts of magnetic field parameter on the flow problem. In addition, enlargement of permeability yields the increment of both temperature distribution and volumetric concentration (see Figs. 14 and 15).

**Figure 16:**
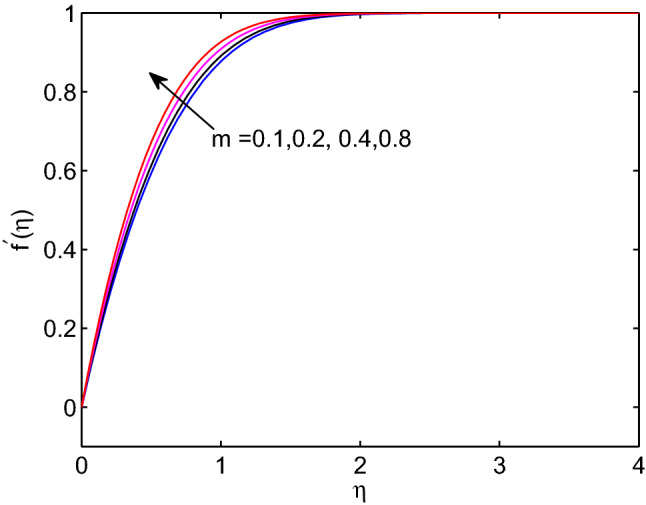
Wedge angle parameter effects on velocity profiles.

Fig. 16 explores influence of wedge angle parameter on tangent hyperbolic nanofluid velocity. Here, augmenting wedge angle parameter produces an enhanced forced convection. This also in turn gives more hyperbolic tangent nanofluid velocity through the boundary layer. Hence, as wedge angle parameter augments, the hyperbolic tangent fluid velocity increases. In addition, the thickness of momentum boundary layer reduces with the increment of wedge angle parameter.

**Figure 17:**
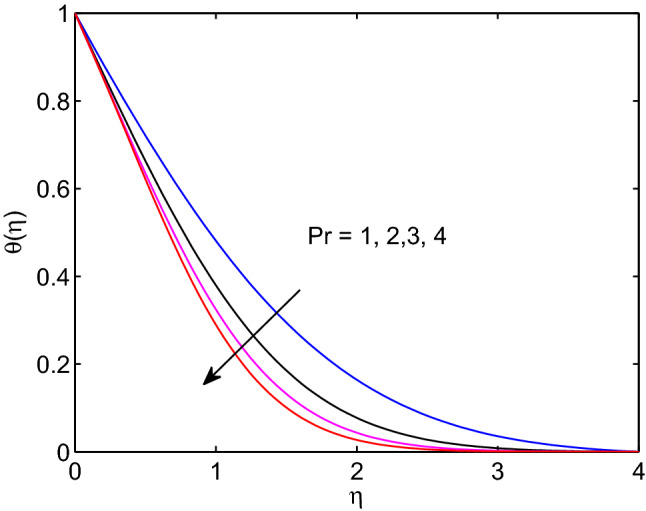
Prandtl number effects on temperature profiles.

**Figure 18:**
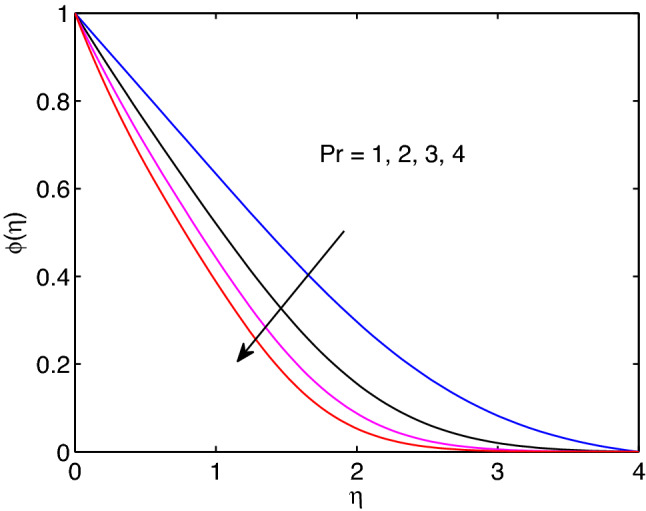
Prandtl number effects on volumetric concentration profiles.

Prandtl number is a non-dimensional quantity occurs in the study of the dynamics of viscous fluids. It can be described as the quotient of viscous force or kinematic viscosity to the nanofluid thermal diffusivity. In this line, it measures the relative significance of heat conductivity and fluid viscosity and varies from one fluid to the other by its numerical values. Its effects on fluid temperature distribution and volumetric concentration of the fluid are disclosed in Figs. 17 and 18. Here, both temperature and nanoparticle concentration of the tangent hyperbolic nanofluid including their boundary layer thicknesses diminish with the increment of Prandtl number.

**Figure 19:**
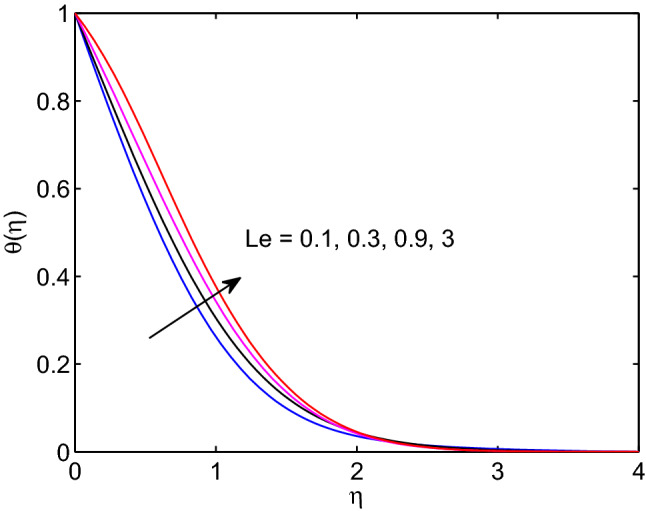
Lewis number effects on temperature profiles.

The physical quantity that can be described as the quotient of thermal diffusivity to the molar diffusivity of the hyperbolic tangent nanofluid is known as Lewis number. Due to this the tangent hyperbolic nanofluid temperature distribution with its boundary layer thickness elevates with the elevation of Lewis number as explored in Fig. 19. However, the volumetric concentration of hyperbolic tangent nanofluid and its boundary layer thickness diminish with enlargement of this number as explored above figures.

**Figure 20:**
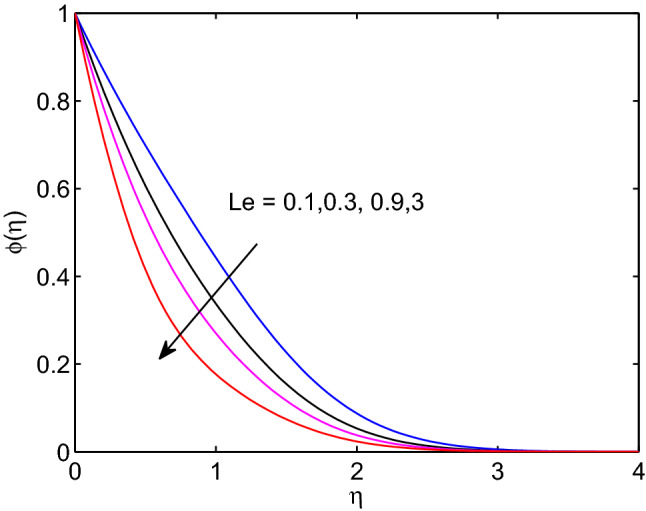
Lewis number effects on volumetric concentration profiles.

**Figure 21:**
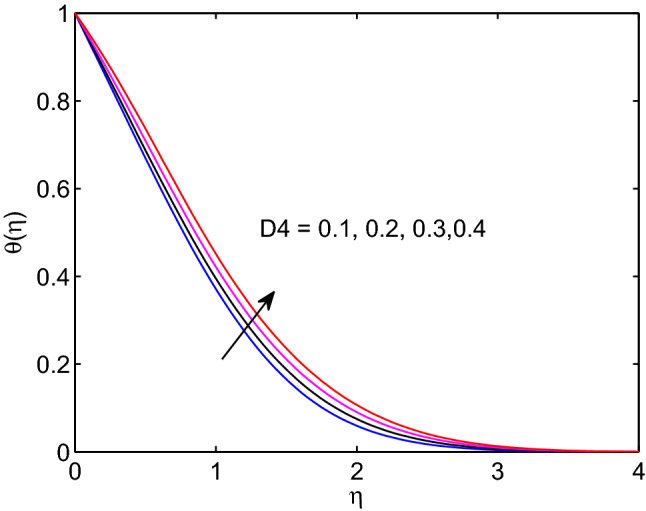
Dufour number effects on temperature profiles.

**Figure 22:**
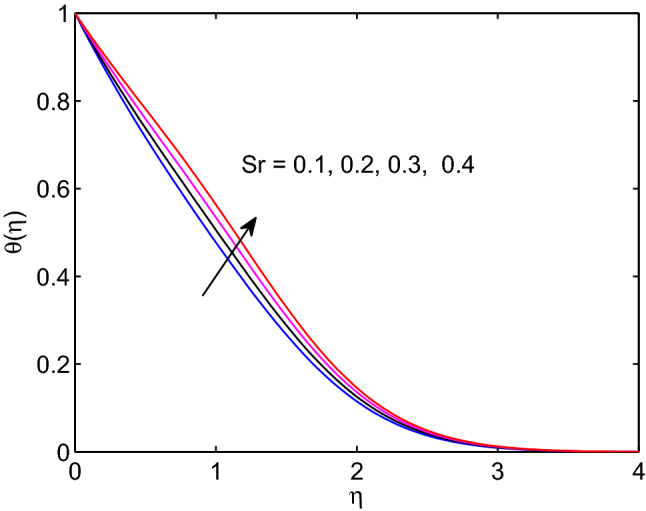
Soret number effects on volumetric concentration profiles.

The Dufour number effect on temperature of hyperbolic tangent nanofluid is given in Fig. 21. Here, one can observe that as Dufour number elevates, the tangent hyperbolic nanofluid temperature distribution as well the thermal boundary layer thickness expand through the flow system. In fact, Dufour number can be defined as the energy flux owing to volumetric concentration gradient and presenting a coupled form of non-reversible processes. Soret number generates more energy flux owing to the temperature difference in the equation of volumetric concentration. Due to this volumetric concentration of tangent hyperbolic nanofluid expands with its boundary layer thickness as Soret number increases as explored in Fig. 22.

**Figure 23:**
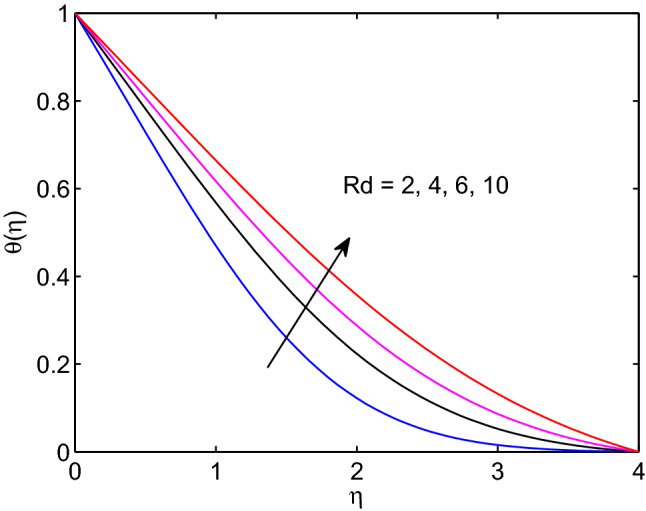
Thermal radiation effects on temperature profiles.

**Figure 24:**
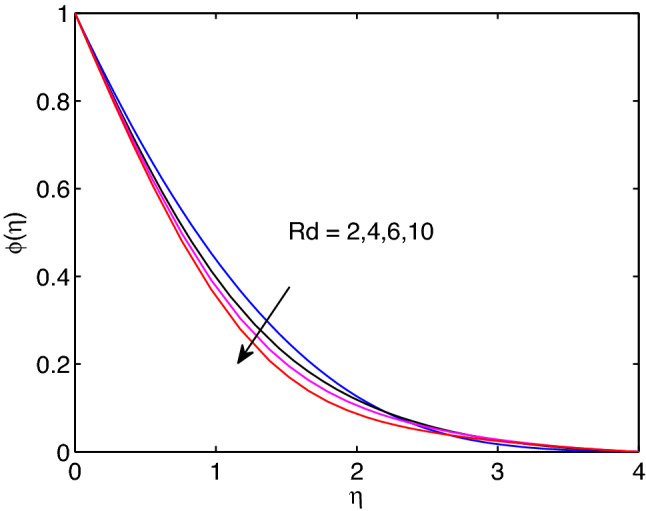
Thermal radiation effects on volumetric concentration profiles.

**Figure 25:**
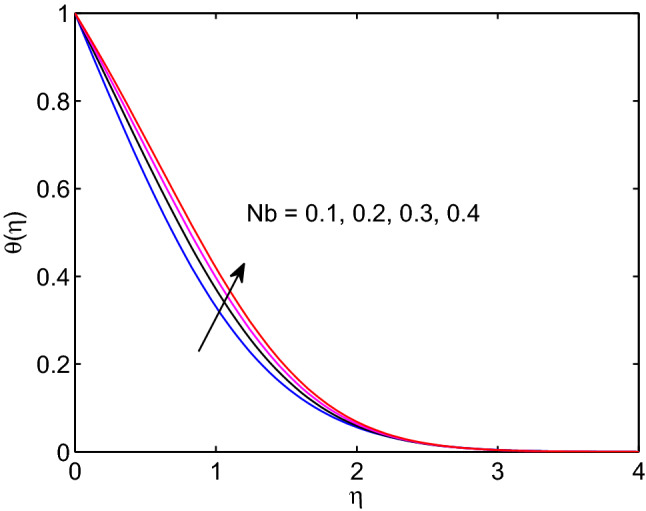
Brownian motion effects on temperature profiles.

**Figure 26:**
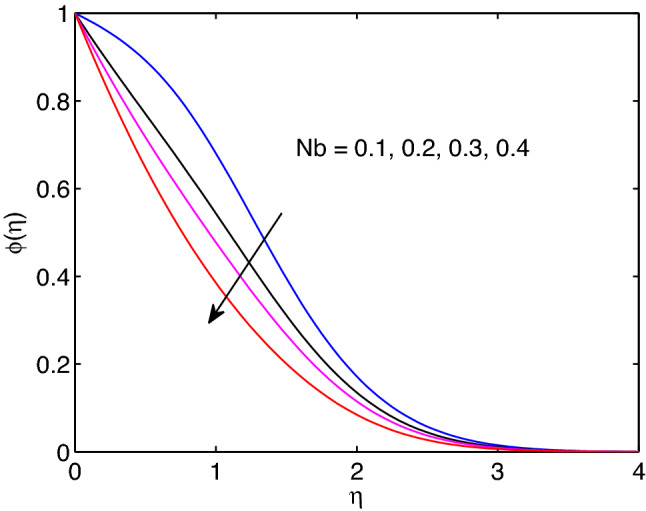
Brownian motion effects on volumetric concentration profiles.

**Figure 27:**
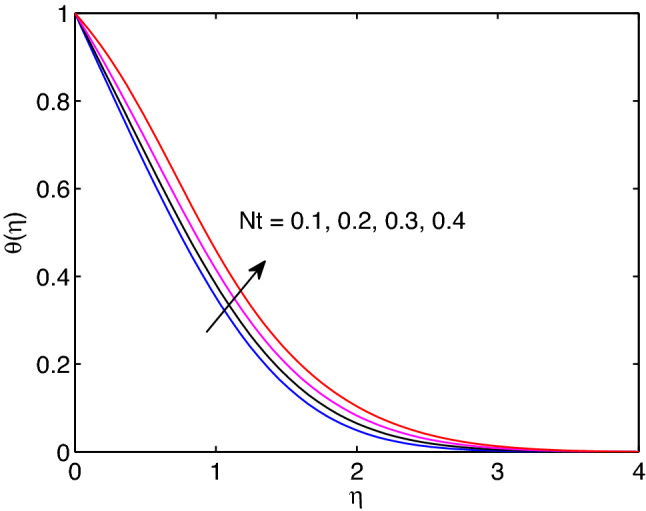
Thermophoresis effects on temperature profiles.

**Figure 28:**
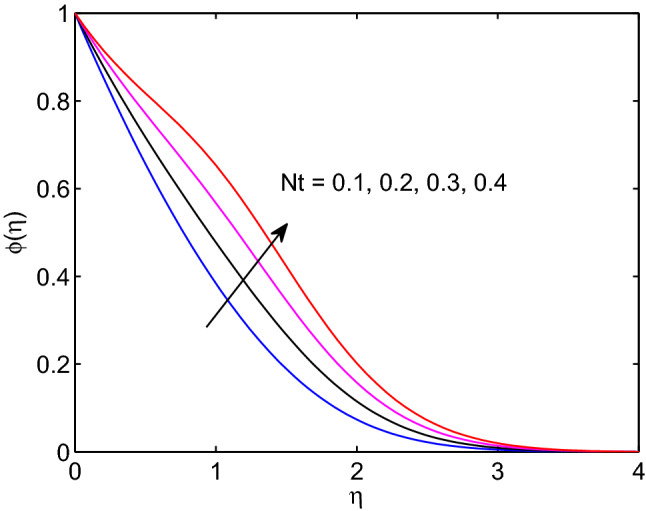
Thermophoresis effects on volumetric concentration profiles.

Thermal radiation entity effects on tangent hyperbolic nanofluid temperature and volumetric concentration is explored in Figs. 23 and 24, respectively. In fact, thermal radiation completely relies on surrounding temperature as it can be emitted in the form of electromagnetic waves. From this we can say that thermal radiation is a function of temperature. Hence, as it gets rise in its numerical values, the tangent hyperbolic nanofluid temperature distribution including its thickness of boundary layer expand together. However, the volumetric concentration including its boundary layer thickness reduce with the rise of thermal radiation entity.

A physical phenomena in which some quantities are regularly undergoing small and arbitrary fluctuations can be termed as Brownian motion. In this process, there is an attack or collision of particles in motion through the flow system. This may result in augmenting of hyperbolic tangent nanofluid temperature and a reduction of volumetric concentration in the regime of flow with increase of Brownian motion as shown in Figs. 25 and 26.

Figs. 27 and 28 represent the influences of thermophoresis parameter on hyperbolic tangent nanofluid temperature and nanoparticle concentration. When we look at these figures, we can realize that both temperature distribution and volumetric concentration along with their thicknesses of boundary layers enlarge with the augmentation of thermophoresis parameter. The concept of physics tells us that this parameter can be expressed as small particle in motion towards positive temperature of gradient. Due to this physical fact temperature distribution and nanoparticle concentration expand with the rise of thermophoresis parameter.

**Table 3:** Variation of Shearing stress, heat and mass transfer rates.

*m*	*Wi*	*n*	*Pr*	*Nb*	*Nt*	D4	Sr	M	*Le*	*B*	Rd	$f''(0)$	$-\theta '(0)$	$-\phi '(0)$
**0.5**	0.5	0.5	3	0.2	0.2	0.1	0.1	0.1	0.5	1	1	3.37290	0.72655	0.67551
**0.6**	0.5	0.5	3	0.2	0.2	0.1	0.1	0.1	0.5	1	1	3.38565	0.72706	0.67581
0.5	**0.5**	0.5	3	0.2	0.2	0.1	0.1	0.1	0.5	1	1	3.37290	0.72655	0.67551
0.5	**0.6**	0.5	3	0.2	0.2	0.1	0.1	0.1	0.5	1	1	3.23984	0.72279	0.67346
0.5	0.5	**0.5**	3	0.2	0.2	0.1	0.1	0.1	0.5	1	1	3.37290	0.72655	0.67551
0.5	0.5	**0.6**	3	0.2	0.2	0.1	0.1	0.1	0.5	1	1	3.36002	0.72851	0.67698
0.5	0.5	0.5	**3**	0.2	0.2	0.1	0.1	0.1	0.5	1	1	3.37290	0.72655	0.67551
0.5	0.5	0.5	**4**	0.2	0.2	0.1	0.1	0.1	0.5	1	1	3.34550	0.71362	0.82602
0.5	0.5	0.5	3	**0.2**	0.2	0.1	0.1	0.1	0.5	1	1	3.37290	0.72655	0.67551
0.5	0.5	0.5	3	**0.3**	0.2	0.1	0.1	0.1	0.5	1	1	3.36144	0.64663	0.81191
0.5	0.5	0.5	3	0.2	**0.2**	0.1	0.1	0.1	0.5	1	1	3.37290	0.72655	0.67551
0.5	0.5	0.5	3	0.2	**0.4**	0.1	0.1	0.1	0.5	1	1	3.36392	0.66391	0.45893

0.5	0.5	0.5	3	0.2	0.2	**0.1**	0.1	0.1	0.5	1	1	3.37290	0.72655	0.67551
0.5	0.5	0.5	3	0.2	0.2	**0.5**	0.1	0.1	0.5	1	1	3.32061	0.36064	0.92273
0.5	0.5	0.5	3	0.2	0.2	0.1	**0.1**	0.1	0.5	1	1	3.37290	0.72655	0.67551
0.5	0.5	0.5	3	0.2	0.2	0.1	**0.5**	0.1	0.5	1	1	3.38050	0.77949	0.41864
0.5	0.5	0.5	3	0.2	0.2	0.1	0.1	**0.1**	0.5	1	1	3.37290	0.72655	0.67551
0.5	0.5	0.5	3	0.2	0.2	0.1	0.1	**0.4**	0.5	1	1	3.40690	0.72769	0.67616
0.5	0.5	0.5	3	0.2	0.2	0.1	0.1	0.1	**0.5**	1	1	3.37290	0.72655	0.67551
0.5	0.5	0.5	3	0.2	0.2	0.1	0.1	0.1	**0.8**	1	1	3.36253	0.65426	1.05210
0.5	0.5	0.5	3	0.2	0.2	0.1	0.1	0.1	0.5	**1**	1	3.37290	0.72655	0.67551
0.5	0.5	0.5	3	0.2	0.2	0.1	0.1	0.1	0.5	**2**	1	3.58278	1.08373	0.77493
0.5	0.5	0.5	3	0.2	0.2	0.1	0.1	0.1	0.5	1	**1**	3.37290	0.72655	0.67551
0.5	0.5	0.5	3	0.2	0.2	0.1	0.1	0.1	0.5	1	**2**	3.35259	0.58477	0.74551

Significant values are in [bold].

Emerging important physical entities influencing surface shearing stress, surface heat transfer coefficient together with volumetric mass transfer gradient are supplied on Table 3. From this table one can realize that shearing stress elevates with the augmentation of *m*, *Sr*, *M*, *B* and *Rd*. However, it diminishes with rise of *Wi*, *n*, *Pr*, *Nb*, *Nt*, *D*4 and *Le*. In this table, we can see also heat transfer rate in magnitude elevates with elevation of *m*, *n*, *Sr*, *M* and *B* whereas it reduces with enlargement of *Wi*, *Pr*, *Nb*, *Nt*, *Rd*, *D*4 and *Le*. Moreover, the magnitude of Sherwood number increases with increase of *m*, *n*, *Pr*, *Nt*, *M*, *D*4, *B*, *Rd* and *Le* whereas it reduces as *Wi*, *Nt* and *Sr* increase. Most importantly, it is visualized that shearing stress, magnitude of heat transfer rate and magnitude of volumetric mass transfer gradient diminish as Weissenberg number increases.

## Conclusions

In this manuscript we have investigated the combined effects of melting process and wedge angle entity on the hydromagnetic hyperbolic tangent nanofluid flow over a permeable wedge under thermal radiation, and, Soret and Dufour effects. Computational results for hyperbolic tangent nanofluid velocity, temperature distribution, and volumetric concentration together-with surface velocity gradient, surface temperature, and surface volumetric gradients are obtained numerically and are each result is with physical rationale. The major findings of the current investigation are drawn as follows:

- Hyperbolic tangent nanofluid velocity reduces with the increase of Weissenberg number whereas the momentum boundary layer thickness augments with elevation of Weissenberg number.
- All the shearing stress, the gradient of surface heat transfer and volumetric mass transfer gradient diminish as Weissenberg number increases.
- The increase of Weissenberg number causes the enlargement of thermal and solutal boundary layer thicknesses .
- The momentum, solutal and thermal boundary layers thickness diminish with expansion of melting heat transfer.
- As wedge angle parameter and power law index elevate, both hyperbolic tangent nanofluid velocity as well as its thickness of boundary layer decrease.
- Increment of Lewis number results the increment in temperature distribution and its thickness of boundary layer whereas a reduction in volumetric concentration as well as its boundary layer thickness.
- The increase of temperature distribution and its boundary layer thickness elevate with elevation of both diffusion-thermo and thermo-diffusion effects.
- The thickness of thermal boundary layer and temperature distribution augment with elevation of thermal radiation parameter.
- As Brownian motion increases, the hyperbolic tangent nanofluid temperature along with its boundary layer thickness increase whereas the volumetric concentration and its boundary layer thickness decrease.
- An increment in thermophoresis causes an increment in both temperature and volumetric concentration.

It is recommended that this investigation can be studied by incorporating Hall current effects in the momentum equation and dissipative terms in the energy equation and in the future work. The Hall current effects and dissipative terms play significant roles in various engineering and science applications.

## Data Availability

We declare that the materials described in the manuscript, including all relevant raw data, will be freely available to any scientist wishing to use them for non-commercial purposes, without breaching participant confidentiality. Further, the corresponding author (M.F.E) should be contacted if someone wants to request the data from this study.
